# Paracellular route is the major urate transport pathway across the blood‐placental barrier

**DOI:** 10.14814/phy2.12013

**Published:** 2014-05-20

**Authors:** Ichiro Uehara, Toru Kimura, Shinji Tanigaki, Toshiyuki Fukutomi, Keiji Sakai, Yoshihiko Shinohara, Kimiyoshi Ichida, Mitsutoshi Iwashita, Hiroyuki Sakurai

**Affiliations:** 1 Department of Obstetrics and Gynecology, Kyorin University School of Medicine, Mitaka, Tokyo, Japan; 2 Department of Pharmacology and Toxicology, Kyorin University School of Medicine, Mitaka, Tokyo, Japan; 3 Department of Pathophysiology, Tokyo University of Pharmacy and Life Sciences, Hachiouji, Tokyo, Japan

**Keywords:** Paracellular, placenta, syncytiotrophoblast, urate transporter, uric acid

## Abstract

Urate, the final oxidation product of purine metabolism, is excreted into urine in humans. Clinically, increased serum urate levels are indicative of pregnancy‐induced hypertension (PIH). However, how urate is handled in the placenta is still largely unknown. In this study, we compared maternal serum urate levels with those of umbilical cord blood and investigated urate transport mechanisms in BeWo cells, a trophoblast‐derived cell line. The maternal and umbilical cord blood samples and placentas were collected from patients undergoing cesarean section at Kyorin University Hospital after obtaining informed consents. There were no significant differences in serum urate levels between maternal blood and umbilical cord blood, and between umbilical cord vein and arterial blood, suggesting that urate is freely movable at the placenta and that fetus is not a major source of urate production. RT‐PCR and immunohistochemistry showed that urate transporters including OAT4, OAT10, GLUT9/URATv1 and ABCG2 were expressed in the syncytiotrophoblast cells in the placenta as well as BeWo cells. Despite expressing aforementioned urate transporters BeWo cells did not take up urate. After confirming the formation of tight junctions of these cells cultured on the transwell, urate transport between upper and lower chambers was measured. Urate moved through BeWo cell monolayers with nonsaturation kinetics and this movement was observed even when the cells were incubated at 4°C, suggesting that urate moves through the paracellular route by simple diffusion.

## Abstract (precis)

Serum urate concentration was identical between mother and fetus, indicating that urate can pass through blood‐placental barrier. Using trophoblast‐derived BeWo cell monolayer, this study demonstrated that urate moved through BeWo cell monolayer via paracellular route.

## Introduction

Urate is a final metabolic product in humans and ~70% of urate is excreted by the kidney. Serum urate concentrations in pregnant women decrease at the first trimester by at least 25%, and returned to levels in nonpregnant women between middle pregnancy and late pregnancy (Boyle et al. 1966) possibly due to increased extracellular volume and glomerular filtration rate during early pregnancy. It has also been known that maternal serum urate concentrations rise in multiple pregnancy (Fischer et al. 1995, 2001; Cohen et al. 2002; Suzuki and Yoneyama 2004), suggesting that the presence of fetus and/or placenta contributes to the increased production of urate. In addition, hyperuricemia is one of the characteristic findings in pregnancy‐induced hypertension (PIH) (Slemons and Bogert 1917; Liedholm et al. 1984; Fievet et al. 1985; Voto et al. 1988; Kabi et al. 1994; Wakwe and Abudu 1999) and frequently precedes the development of PIH. Thus, hyperuricemia has been used as an early warning sign of PIH (Liedholm et al. 1984; Voto et al. 1988; Wakwe and Abudu 1999) among pregnant women. In PIH, compromised renal function may explain hyperuricemia. We attempted to investigate urate handling in pregnancy‐associated hyperuricemia and noticed that there has been no knowledge about how urate moves across the blood‐placental barrier.

In the kidney, urate is filtered through glomerulus followed by reabsorption and secretion that takes place in the proximal tubule (Anzai et al. 2007, 2012; Wright et al. 2010; Bobulescu and Moe 2012; Lipkowitz 2012). We and others have shown several transporters including SLC22A12 (urate transporter 1, URAT1), SLC2A9 (glucose transporter 9, GLUT9/voltage‐dependent urate transporter 1, URATv1), SLC22A11 (organic anion transporter 4, OAT4), SLC17A1/3 (sodium phosphate transporter 1/4, NPT1/4), and ATP‐binding cassette subfamily G member 2 (ABCG2) play an important role in urate reabsorption or secretion. SLC2A9 has been reported to be expressed in the placenta and we initially hypothesized that some of above‐mentioned urate transporters are also responsible for urate movement across the placenta. However, as shown in this manuscript, urate crossed the placental epithelia, syncytiotrophoblast cells, via the paracellular route.

## Materials and Methods

### Chemicals

Glibenclamide, an ABCG2 inhibitor, benzbromarone, an OATs inhibitor, and fumitremorgin C, an MRP4 inhibitor were purchased from Wako Pure Chemical Industries, Ltd (Osaka, Japan). Radioactive [8‐^14^C]‐Uric acid and [2.8‐^3^H]‐Hypoxanthine were purchased from Moravek Biochemicals (Brea, CA). [6,7‐3H(N)]‐estrone 3‐sulfate ammonium salt was purchased from American Radiolabeled Chemicals Inc. (St Louis, MO). Fluorescein‐conjugated dextran (MW; 3000) was obtained from Life Technologies (Carlsbad, CA).

### Antibodies

Isoform‐specific anti‐GLUT9/URATv1 antibodies were produced in rabbit, as described elsewhere (Kimura et al. 2014). Anti‐OAT4 antibody was produced previously (Cha et al. 2000). Anti‐ ABCG2 antibody was obtained from Merck (Darmstadt, Germany) and anti‐OAT10 antibody was obtained from Sigma (St. Louis, MO). Anti‐ platelet endothelial cell adhesion molecule‐1 (PECAM‐1) and anti‐human placental alkaline phosphatase (PLAP) antibodies were from Cell Signaling (Danvers, MA) and Millipore (Billerica, MA), respectively.

### Specimens

Placental tissues, maternal and umbilical cord blood were obtained from patients undergoing cesarean section at 26–41 weeks of gestation in Kyorin University Hospital. There were 50 women with normal singleton pregnancy, 21 women with twin pregnancy, and 13 women with PIH. Patients with chronic hypertension, renal disease, and other systemic disease were excluded. All patients gave informed consent for collection and investigational use of tissues. This study protocol was approved by the ethics committee of Kyorin University School of Medicine.

### Measurement of urate concentration

Maternal blood was obtained on the day before or in the morning of delivery and paired umbilical cord blood was collected from umbilical artery and vein at delivery. After the sample is centrifuged for 10 min at 1700 *g*, the supernatant was collected and stored at −80°C until measurement. Urate concentration was measured at Nagahama Life Science Laboratory (Shiga, Japan).

### Measurement of hypoxanthine concentration

Perchoric acid was added to serum samples and centrifuged to remove serum proteins. The supernatant was filtrated with 0.45‐*μ*m membrane filter. The quantitative analysis of hypoxanthine was performed using High Performance Liquid Chromatography with UV detection at 254 nm with an ODS column (140 × 4.6 mm, 5 *μ*m, Mighysil RP‐18 GA Aqua; Kanto chemical co., Inc. Tokyo, Japan) using water containing 0.1% TFA as mobile phase.

### Detection of urate transporters expression by RT‐PCR

Placental tissues were immediately cut into small pieces and snap‐frozen in liquid nitrogen and then stored at −80°C. Isolation of total RNA from the placenta and BeWo cells; a trophoblast‐derived epithelial cancer cell line was performed with ISOGEN (NIPPON GENE, Tokyo, Japan) according to the product manual. After extracting RNA, the samples were treated with DNase to remove genomic DNA. RNA was purified by extraction with phenol, chloroform, and isoamyl alcohol and ethanol precipitation. Reverse transcription was performed with Superscript III First‐Strand Synthesis System (Life Technologies) according to the product manual. PCR reaction was performed with two or three kinds of primer pairs for each transporter (Table 1) using Gene Amp PCR 9700 (Life Technologies).

**Table 1.** Primer pairs used for RT‐PCT

Transporters	Primer sequence	Product size (bp)
URAT1		
F1	AACCTCGTGTGTGACTCT	414
R1	AAAGCAGAGGAAGAAGGG	
F2	CCCTTCTTCCTCTGCTTT	370
R2	ATGTCCACGACACCAATG	
NPT1		
F1	CCAGATATCCAGGGAATC	416
R1	AGAAGACATACGGCACAG	
F2	GCAGGTCAGTTCAAGTAG	390
R2	GCACCAGCAAGTATTAGG	
NPT4		
F1	GCCCCAAAGAGTCTTCCTGC	505
R1	TCCATGGATAGGAAACGG	
F2	CTTCTAAGCAGCCTCTTC	372
R2	AAGAGAGCGTCAGCAAGGCA	
OAT1		
F1	CCGGAAGGTACTCATCTT	334
R1	GGCCGACTCAATGAAGAA	
F2	TTGTCATCAACTCCCTGG	481
R2	TGGTGCTCTTGTTGCTGT	
OAT3		
F1	GATCCTCAACATGGCCAA	331
R1	CCCTCCAATCAGTATACC	
F2	GGAGGAGCTCAAACTCAA	419
R2	TTGTGTAGAGGAAGAGGC	
OAT4		
F1	TTTCTGAGTTCACTGACACT	387
R1	CACGCAGAACAGGTCCAGCA	
F2	GTTCTCGAAGCTCTTGGA	445
R2	CATGAAGATGGACTGGCT	
OAT10		
F1	CCCATCCCTGAAGAATGA	470
R1	AACGTGCAGATTCTGGCA	
F2	AGCTGGTCCCAGAGAAGAC	480
R2	TGGTGTGAGGATGCCCCCGA	
URATv1		
F1	ATGAAGCTCAGTAAAAAGGAC	230 (short isoform)
R1	GAGTGTCTGGGTCTATTGGA	
F2	ACTGAGACCCATGGCAAGGAAA	326 (long isoform)
R2	GAGTGTCTGGGTCTATTGGA	
F3	GGCCTCAATGCAATTTGG	373
R3	CTGCAATGATGAAGGCAG	
ABCG2		
F1	GCTGCAAGGAAAGATCCA	509
R1	CTTCCTGAGGCCAATAAG	
F2	GCAGGATAAGCCACTCAT	432
R2	GACACTCTGTAGTATCCG	
MRP4		
F1	TACCAGGAGGTGAAGCCCAA	555
R1	TGTCTTCCCCATGGCCATGT	
F2	AGTTGGTTTTCAGGCCTATA	473 (variant1)
R2	CACCAACCACTTGTAGCAAT	
F3	AGTTGGTTTTCAGGCCTATA	472 (variant2)
R3	CAACACAGCGAGATCCCATC	
F4	TGGTGTGTTCGACAAAGTGC	469 (variant1)
R4	GTAAGGCATTCCACAGTTCC	

### Immunohistochemistry

To examine the distribution of transporters within the placenta, tissue sections were stained with TSA Fluorescence System (PerkinElmer, Inc., Waltham, MA) according to the manufacturer's instructions with minor modifications (Zhao et al. 2003). In brief, sections were blocked with TNB buffer (0.1 mol/L Tris‐HCl, pH 7.5, 0.15 mol/L NaCl and 0.5% Blocking Reagent) containing 5% normal donkey serum. To block endogenous biotin, the sections were further treated with the Avidin–Biotin block (Vector Laboratories, Burlingame, CA), and endogenous peroxidase activity was quenched with 1% H_2_O_2_. The primary antibody was applied overnight at 4°C. Slides were washed with TNT Wash Buffer (0.1 mol/L Tris‐HCl, pH 7.5, 0.15 mol/L NaCl, and 0.05% Tween20) and incubated with biotin‐conjugated donkey anti‐rabbit IgG (Jackson ImmunoResearch Laboratories, Inc., West Grove, PA), followed by incubation with streptavidin–HRP conjugate (Life Technologies) together with 1 *μ*g/mL DAPI. The antigens were detected using tyramide‐FITC or tyramide‐Cy3. Sections were mounted in Fluorescence Mounting Medium (Dako, Glostrup, Denmark). Fluorescence was visualized with an Olympus Fluoview FV1000 laser confocal microscope (Tokyo, Japan). Images were the product of threefold line averaging. Contrast and brightness settings were chosen so that all pixels were within the linear range.

### Cell culture

BeWo cells (American Type Culture Collection (ATCC), Manassas, VA), a trophoblast‐derived epithelial cancer cells were cultured in a humidified incubator under 5% CO_2_ in Nutrient Mixture F‐12 Ham Medium (Sigma‐Aldrich, St. Louis, MO) supplemented with 10% FBS, 2 mmol/L l‐Glutamine, 50 U/mL penicillin, and 50 *μ*g/mL streptomycin. COS‐7 cells and MDCK cells (ATCC) were cultured in a humidified incubator under 5% CO_2_ in Dulbecco's Modified Eagle's Medium (Sigma‐Aldrich) supplemented with 10% FBS, 2 mmol/L l‐Glutamine, 50 U/mL penicillin, and 50 *μ*g/mL streptomycin.

### Urate, hypoxanthine, and estrone sulfate up take

BeWo cells were seeded on 24‐well plates and grown to more than 90% confluence. Cells were then washed twice with pre‐warmed uptake solution (125 mmol/L NaCl, 4.8 mmol/L KCl, 1.2 mmol/L CaCl_2_, 1.2 mmol/L KH_2_PO_4_, 1.2 mmol/L MgSO_4_, 5.6 mmol/L D‐glucose, and 25 mmol/L HEPES, pH 7.4). Cells were incubated with uptake solution containing 4 *μ*mol/L of radioactive urate, 0.1 *μ*mol/L of radioactive hypoxanthine or 0.02 *μ*mol/L of radioactive estrone sulfate and their final concentrations were 200, 5, and 10 *μ*mol/L, respectively, after adding cold molecules. Cells were incubated for indicated times at 37 and 4°C (control) and then uptake of substrates was terminated by washing cells with ice‐cold uptake solution three times. Cells were solubilized in 0.1 N sodium hydroxide and radioactivity of cell lysates was measured by a liquid scintillation counter (LSC‐5100, Hitachi Aloka Medical, Tokyo, Japan).

### Confirmation of tight junction formation

Tight junction formation was confirmed by measurement of transepithelial electrical resistance (TER) and permeability of 3000 dalton (Da) dextran. Cells were grown to confluence on the cell culture inserts (BD, Franklin Lake, NJ). TER was measured with EVOM2, Epithelial Voltohmmeter for TEER and Chopstick Electrode, STX2 (World Precision Instruments, Sarasota, FL). For permeability of 3000 Da dextran, cells were grown to confluence on the cell culture inserts. Cells were kept on ice and washed with cold uptake solution three times. About 650 *μ*L of uptake solution was added to the bottom wells and 230 *μ*L of uptake solution containing 10 *μ*mol/L fluorescein‐conjugated 3000 Da dextran was added to the top wells of the cell culture inserts. After the cells were incubated for indicated times at 4°C medium in the bottom wells was obtained and fluorescence was measured by fluorescence microplate reader to determine leakage of fluorescein‐conjugated dextran from the top to the bottom wells.

### Measurement of paracellular urate movement

Cells were grown on the culture inserts and confirmed tight junction formation described above. Cells were washed with ice cold uptake solution and incubated with uptake solution containing 4 *μ*mol/L [^14^C]‐urate plus 196 *μ*mol/L cold urate from the top or bottom wells at 4°C. When urate concentration dependency was measured, final urate concentration was adjusted with 4 *μ*mol/L [^14^C]‐urate and cold compound. After incubation for indicate time, radioactivity in the opposite side was measured as urate paracellular movement.

## Results

### Urate concentration of maternal and fetal blood

**Figure 1.**
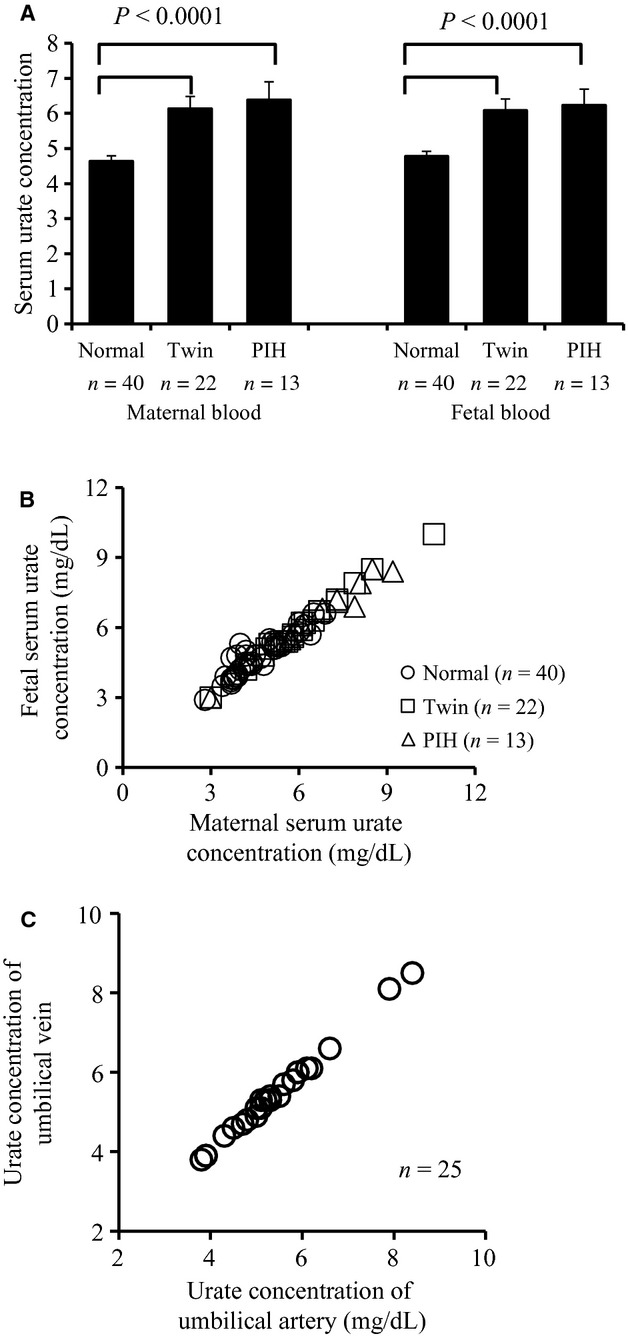
Serum urate concentrations of mother and fetus. (A) The columns represent the mean ± SD of serum urate levels (mg/dL) in maternal blood (left panel) and umbilical cord blood (right panel) obtained from normal singleton pregnancies, twin pregnancies, and pregnancies with PIH. (B) Comparative analysis between serum urate levels in paired maternal and umbilical cord bloods is shown. Open circles, squares, and triangles represent normal singleton pregnancies, twin pregnancies, and pregnancies with PIH, respectively. Correlation coefficient was 0.881, *P* < 0.001. (C) Comparative analysis between serum urate levels in paired arterial and venous bloods of umbilical cord is shown. Correlation coefficient was 0.992, *P* < 0.001.

Serum urate concentration of maternal and fetal blood in normal singleton, twins, and PIH pregnancies were measured (Fig. 1A). As reported previously, maternal urate levels were significantly higher in twin pregnancies or PIH patients compared to those of normal singleton pregnancies (6.14 ± 0.34, 6.39 ± 0.51 mg/dL vs. 4.64 ±0.15 mg/dL, *P* < 0.0001). Not only maternal urate levels but also fetal urate levels were higher in twin pregnancies or pregnancies with PIH compared to those of normal singleton pregnancies (6.09 ± 0.32, 6.24 ± 0.45 mg/dL vs. 4.78 ± 0.14 mg/dL, *P* < 0.0001).

Next, paired maternal and fetus urate concentrations were compared (Fig. 1B). Interestingly, there were no difference in urate levels between a fetus and its mother or fetuses and their mother in each group and no difference between fetuses in twin pregnancy (data not shown). When all samples from singleton pregnancy, twin pregnancy, and PIH were compared, urate levels between fetus and mother were tightly correlated and virtually identical as indicated in Figure 1B (*r* = 0.881, *P* < 0.001).

In individual cord blood, urate concentration in artery and vein were compared (Fig. 1C). If there is urate production in the fetus or urate excretion into amniotic fluid, urate levels of artery and vein should be different. Surprisingly, urate level in paired artery and vein bloods were almost identical (*r* = 0.999, *P* < 0.001, Fig. 1C), indicating that there is little effect of urate production and excretion in the embryo on maternal serum urate concentration.

### Expression of urate transporters in the placenta

**Figure 2.**
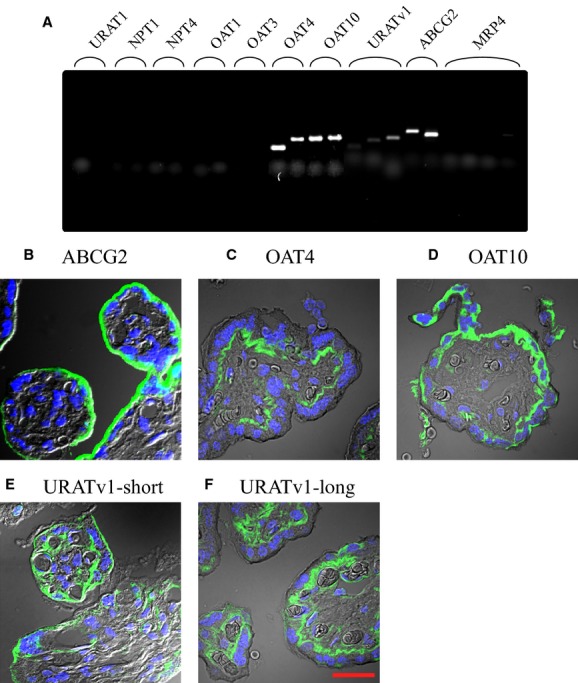
Expression and localization of putative urate transporters. (A) RT‐PCR of urate transporters was performed using placental RNA. Expression of each transporter was analyzed using two or three different primer sets listed in Table 1. (B) Immunofluorescence analysis of the placental tissue were carried out using specific antibodies against urate transporters (green); ABCG2 (B), OAT4 (C), OAT10 (D), URATv1‐short isoform (E), and URATv1‐long isoform (F). Nuclei were visualized by DAPI (blue), and merged pictures with phase contrast are shown. The scale bar of 30 *μ*m is shown in F.

It seems that urate passes placental barrier freely because there is no difference between urate levels of maternal and fetal blood (Fig. 1). Since syncytiotrophoblast cells form the placental barrier, it would be important which urate transporters are expressed in these cells. RT‐PCR was performed using two or three different pairs of specific primers for putative urate transporters (Table 1). As shown in Figure 2A, mRNA for *OAT4*,* OAT10*,* GLUT9/URATv1*, and *ABCG2* were expressed in the placenta but other transporters including *URAT1*,* OAT1*,* OAT3*,* NPT1*, and *NPT4* that are present in the kidney were not expressed. A trophoblast‐derived epithelial cell line, BeWo cells, expressed the same set of transporters as the placenta except that multidrug resistance‐associated protein 4 (*MRP4*) was only expressed in BeWo cells (data not shown).

To confirm their protein expression and localization in the placenta, immunohistochemical staining of the placenta was performed (Fig. 2B–F). Antibodies against ABCG2, OAT4, OAT10, URATv1, whose mRNAs were detected by RT‐PCR were used for the study. It has been reported that the *SLC2A9* gene produces two splice variants (short and long isoforms; URATv1‐short and URATv1‐long) that only differ in their N‐termini (Augustin et al. 2004) and that both isoforms are expressed in the human placenta (Bibee et al. 2011). We produced the antibodies that detect each GLUT9/URATv1 isoform and used them for immunofluorescence (Kimura et al. 2014). Placental alkaline phosphatase (PLAP) was used as a syncytiotrophoblast cell marker and endothelial cell adhesion molecule‐1 (PECAM‐1) was used as an endothelial cell marker. ABCG2 was expressed at the maternal side (brush border membrane) of the syncytiotrophoblast (Fig. 2B), OAT4 was expressed at the embryonic side of the syncytiotrophoblast (basolateral membrane) (Fig. 2C), OAT10 was expressed both at the maternal side and the embryonic side of the syncytiotrophoblast (brush border membrane and basolateral membrane) (Fig. 2D), URATv1‐ short isoform was expressed at the maternal side of the syncytiotrophoblast and endothelial cells (Fig. 2E), and URATv1‐long isoform was expressed at the embryonic side of the syncytiotrophoblast and the endothelial cells (Fig. 2F). These staining patterns of URATv1 were consistent with the previous report (Bibee et al. 2011).

### Urate and hypoxanthine uptake by BeWo cells

**Figure 3.**
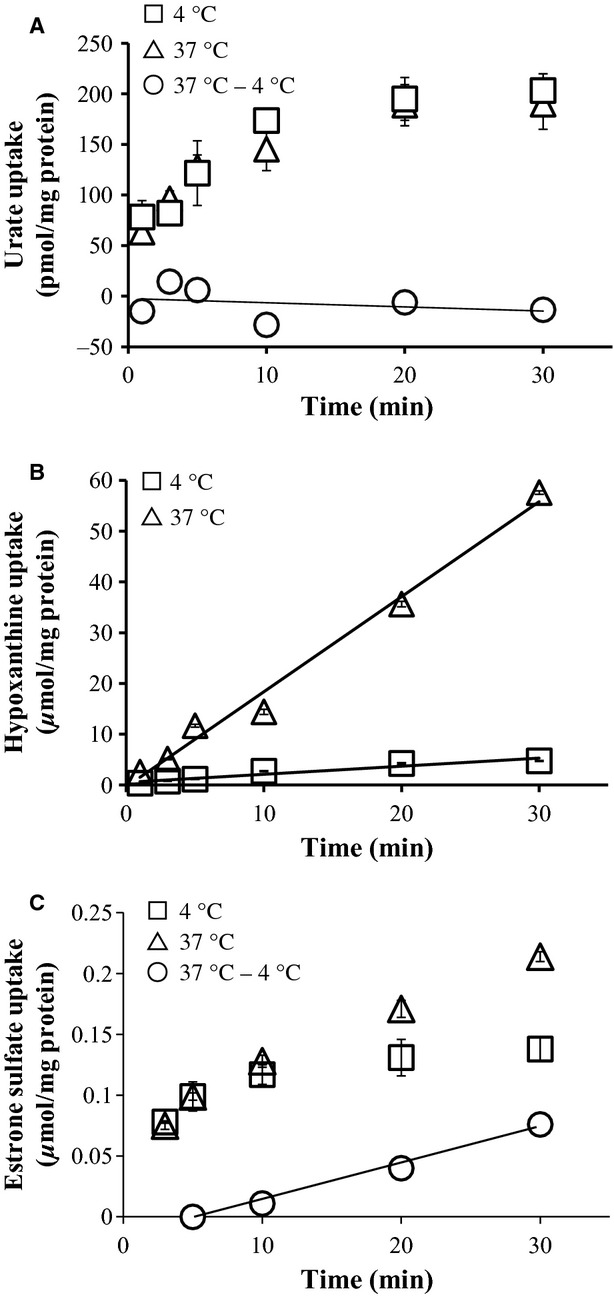
Urate, hypoxanthine, or estrone sulfate uptake by BeWo cells. BeWo cells were incubated with [^14^C]‐urate (A), [^3^H]‐ hypoxanthine (B) or [6,7‐3H(N)]‐estrone 3‐sulfate (C) for indicated times at 37°C (△) or 4°C (□). Difference of measured radioactivity of urate between 37 and 4°C, indicative of net urate uptake, is shown by open circles (○) in A and C.

Since BeWo cells expressed almost the same set of urate transporters as the placenta, these cells were used to assess urate transport function of the placenta in vitro. Radioisotope‐labeled urate uptake was measured both at 37 and 4°C. At 4°C, transporters in the cell are assumed to be inactive and radioactivity measurements reflect nonspecific cell surface adhesion of urate. The net urate uptake, that is radioactivity at 37°C minus that at 4°C, was almost zero throughout the measurement (Fig. 3A). It is possible that this zero uptake is due to urate efflux transporters such as ABCG2 and MRP4. This is unlikely because significant urate uptake was not observed in the presence of fumitremorgin C and glibenclamide, an inhibitor of ABCG2 and MRP4, respectively (data not shown). As [^3^H]‐hypoxanthine was taken up by BeWo cells (Fig. 3B), the lack of urate uptake by the same cell cannot be explained by general incapability of transporters in BeWo cells. Furthermore, estrone sulfate, an organic anion like urate, was also taken up by BeWo cells (Fig. 3C). Among the OATs expressed in the BeWo cells, OAT4 is likely to mediate this uptake because estrone sulfate has been shown to be the substrate of OAT4 (Cha et al. 2000) but not OAT10 (Bahn et al. 2008).

### Paracellular urate movement

**Figure 4.**
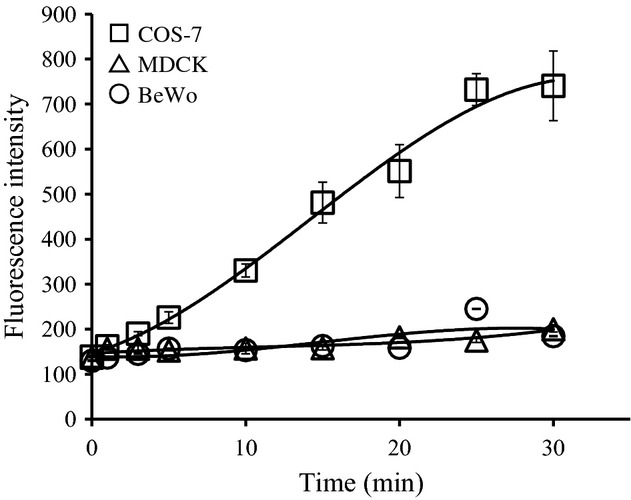
FITC‐dextran (MW 3000) leakage on cell monolayers. Cell lines were cultured on Transwell cell culture inserts to full confluency. Cells were chilled and washed with uptake solution at 4°C and FITC‐dextran was added to upper wells. At indicated times, solution was collected from lower wells and fluorescence was measured.

The paracellular route is one of the other routes for passing though the epithelial cell layer. Whether urate is transported by the paracellular route at placental barrier was examined. TER (Table 2) and permeability of fluorescein isothiocyanate (FITC)‐conjugated 3000 Dalton dextran (Fig. 4) were measured to confirm tight junction formation. TER of COS‐7 cell monolayers, which do not form tight junctions (negative control), was 218Ω. In contrast, BeWo cell monolayers had much higher TER (290Ω) comparable to that of MDCK cell monolayer (289Ω), a prototypical epithelial cell line with tight junctions (positive control). Permeability of fluorescence‐conjugated dextran showed similar results; FITC‐dextran was leaked through COS‐7 cell monolayers but not BeWo or MDCK cell monolayers (Fig. 4). These results indicate that BeWo cells form functional tight junctions as MDCK cells.

**Table 2.** TER value

Cells	TER (Ω)
Empty	146 ± 9.1
BeWo	290 ± 7.2
COS‐7	218 ± 3.0
MDCK	289 ± 6.0

**Figure 5.**
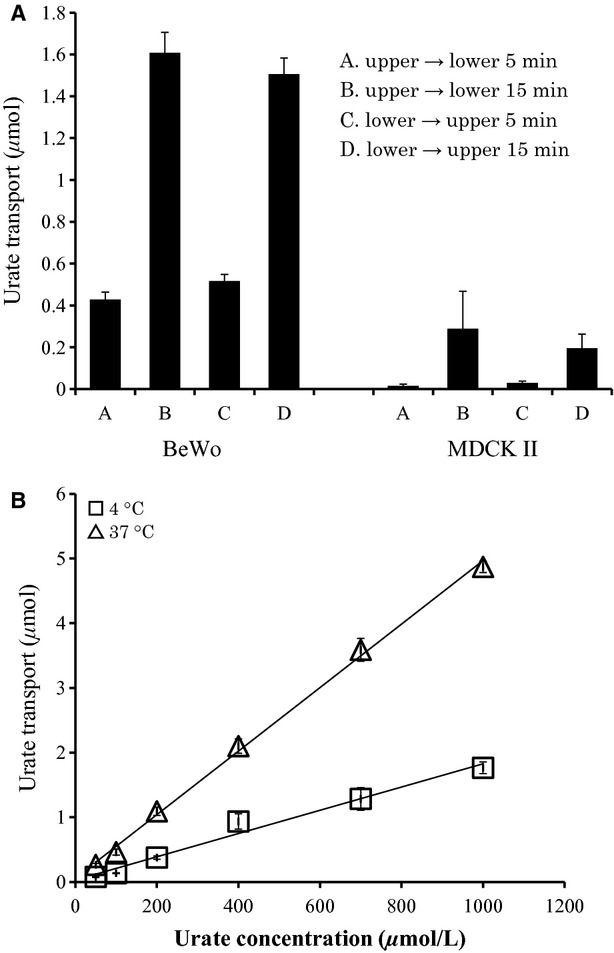
Paracellular transport of urate through cell monolayers. (A) Cell lines were cultured on Transwell cell culture inserts to full confluency. Urate movement was measured at 4°C to avoid influence of transporters and endocytosis/exocytosis. [^14^C]‐urate was added to upper or lower wells and incubated for 5 or 15 min. After incubation, the medium in the opposite wells was collected and radio activities were measured. (B) BeWo cells were cultured on Transwell cell culture inserts to full confluency. [^14^C]‐urate was added to upper wells and incubated for 5 min in the buffer containing indicated urate concentrations. After the incubation, the medium in the lower wells was collected and radio activities were measured.

Paracellular urate transport was measured for 5 and 15 min at 4°C, where transporters should be inactive. As shown in Figure 5A, MDCK cell layer hardly pass urate through the paracellular route in both directions. In contrast, significant urate movement was observed in BeWo cells. No significant amount of urate was found inside BeWo cells during paracellular urate movement (data not shown). This urate paracellular movement was not inhibited by urate transporter inhibitor such as fumitremorgin C, glibenclamide, and benzbromarone (data not shown). Based on these results, it is unlikely that urate movement through BeWo cell monolayers takes the transcellular route. Another evidence for paracellular urate transport was obtained by kinetic studies. As shown in Figure 5B, urate flux was linearly increased as higher concentrations of urate were applied to the upper chambers, consistent with simple diffusion rather than facilitated diffusion mediated by transporters.

## Discussion

In this study, we showed that serum urate levels were identical between fetus and its mother irrespective of normal singleton pregnancies, twin pregnancies or pregnancies complicated by PIH, suggesting that urate can freely move through placental membrane. Although several putative urate transporters were expressed in the syncytiotrophoblast as well as trophoblast‐derived BeWo cells, urate passed through BeWo cell monolayers via the paracellular route.

In 1917, two groups reported that urate levels of fetal and maternal blood were identical and they concluded that urate may pass through placenta by the process of passive diffusion (Kingsbury and Sedgwick 1917; Slemons and Bogert 1917). However, detailed kinetic studies were not done in these papers. We replicated their results here (Fig. 1B). In addition, the fact that urate levels in the umbilical vein and artery were identical indicate that urate production in the fetus does not contribute to a significant extent to urate levels of a feto‐maternal unit.

In the placenta, urate from maternal circulation has to cross the trophoblast layer to reach fetal blood. As uric acid is a weak acid whose pKa is 5.75, almost all urate exists as a negatively charged anion form at physiological pH. Since charged molecule cannot permeate cell membrane, urate movement across the trophoblast layer has to be transcellular by carrier or endocytosis/exocytosis, or paracellular.

To date, it has been reported that 10 transporters, URAT1 (Enomoto et al. 2002), NPT1 (Uchino et al. 2000), NPT4 (Jutabha et al. 2010), OAT1 (Sekine et al. 1997), OAT3 (Bakhiya et al. 2003), OAT4 (Hagos et al. 2007), OAT10 (Bahn et al. 2008), GLUT9/URATv1 (Anzai et al. 2008), ABCG2 (Woodward et al. 2009), and MRP4 (Van Aubel et al. 2005), have an ability to transport urate. When these transporters are overexpressed in oocytes or culture cells, urate is taken up into the cell. In the placenta as well as BeWo cells, OAT4, OAT10, GLUT9/URATv1, and ABCG2 were expressed. Surprisingly urate uptake, however, was not observed in BeWo cells. Primary trophoblast cells established from placenta did not take up urate despite expressing the same set of urate transporters as trophoblast judged by RT‐PCR (I. Uehara, T. Kimura unpubl. obs.). Of these four transporters, net urate uptake should be mediated by OAT4 and/or OAT10, both of which are anion exchangers so that negatively charged urate can move into the cell against unfavorable electric potential in exchange for a counteranion with favorable electrochemical gradient. GLUT9/URATv1 is a voltage‐driven urate transporter that favors its substrate to move from the electrically negative side to the positive side; that is inside the cell to outside the cell. Because this transporter mediates only passive movement, urate uptake can only be observed when urate concentration outside the cell is unreasonably high in order to counter the unfavorable electrical potential difference. ABCG2 expels urate out of the cell but it does not mediate urate uptake. In our hands, BeWo cells never took up urate. The fact that even in the presence of an ABCG2 or URATv1 inhibitor, BeWo cells did not take up urate is consistent with no urate movement into the cell. These results also speak against endocytosis of urate, in which case a significant amount of urate uptake should be observed.

Therefore, we examined whether urate movement was via the paracellular route. Unfortunately, primary trophoblast cells prepared from the placenta did not form tight junctions in vitro (I. Uehara, T. Kimura unpubl. obs.), so we employed monolayers of BeWo cells as a model for the blood‐placental barrier. Significant urate movement was observed at 4°C (Fig. 5), suggesting that urate moves across the trophoblast layer via the paracellular route. Because general cellular processes requiring energy should be minimal, if any, at 4°C, active transport processes or endocytosis/exocytosis are unlikely to play an important role in this urate movement. Interestingly, urate did not cross monolayers of MDCK cells, a kidney tubule‐derived cell line (Fig. 5).

In the human kidney, the transcellular route is the major pathway for urate transport since loss of function mutations in either URAT1 or GLUT9/URATv1 cause renal hypouricemia (Enomoto et al. 2002; Ichida et al. 2004; Anzai et al. 2008; Matsuo et al. 2008; Dinour et al. 2010). In the rat kidney, carrier‐mediated urate transport is reported to be operational in the proximal tubule based on microperfusion studies (Sansom et al. 1981; Weinman et al. 1983). In contrast, it has been demonstrated that urate is transported by the paracellular route in the avian renal proximal tubules based on the fact that there is no urate in the cells during urate movement (Brokl et al. 1994). This is very similar to what we observed in urate movement via BeWo cell monolayers. Ogura et al. (2012) have shown that urate passes through the rat intestine by the transcellular route as well as the paracellular route using Ussing‐type diffusion chambers. Taken together, it seems that urate can move epithelial cell layers via the transcellular, paracellular route or both depending on spices, tissue, and cell types. Further studies are needed to elucidate which factor(s) are important in choosing the route for urate passage or what is the molecular nature for the paracellular route.

In conclusion, we found that urate concentrations of maternal blood, umbilical artery, and vein were almost identical in normal singleton, twin pregnancies or patients with PIH and that urate passed through the blood‐placental barrier via the paracellular route, providing detailed physiological explanation for “passive urate movement” described in 1917 works of literature.

## Acknowledgments

We thank members of the laboratory group for technical assistance, comments, and helpful discussions.

## Conflict of Interest

None declared.
